# Psycho-rheumatic Integration in Systemic Lupus Erythematosus: An Insight into Antibodies Causing Neuropsychiatric Changes

**DOI:** 10.7759/cureus.3091

**Published:** 2018-08-03

**Authors:** Gouthami Nalakonda, Mimsa Islam, Vera E Chukwu, Ahmad Soliman, Rujina Munim, Inas Abukraa

**Affiliations:** 1 Medical Student, Chalmeda Anandrao Institute of Medical Sciences, Karimnagar, IND; 2 Internal Medicine, Sir Salimullah Medical College, Dhaka, USA; 3 Research, University of Benin, Benin City, NGA; 4 Pediatrics, Sohar Hospital, Sohar, OMN; 5 Miscellaneous, Sylhet Mag Osmani Medical College and Hospital, Sylhet, BGD; 6 Faculty of Medicine, Tripoli University, Tripoli, LBY

**Keywords:** sle, sle antibody brain, anti-nmdar, anti ribosomal p, antiphospholipid, rituximab, tweak

## Abstract

The main purpose of this paper is to bring together all the antibodies and markers related to neurological and psychiatric manifestations in systemic lupus erythematosus and also the pharmacology that could help treat these symptoms. Existing research data regarding specific antibodies involved in the disease process and drugs that were being studied was collected and analyzed. After reviewing the studies published by various authors, symptoms were shown to be mainly caused by antibodies against N-methyl-D-aspartate receptor (NMDAR) antibodies, anti-endothelial, anti-ribosomal P, antiphospholipid antibodies, cytokines like interferons and chemokines. The monoclonal antibody rituximab has shown to be beneficial in some of the cases. Based on all the articles reviewed, the antibodies and cytokines showed the most effective evidence in causing the different manifestations of neuropsychiatric systemic lupus erythematosus (NPSLE), but studies regarding the drugs being effective against all the symptoms are inconclusive as there are very few studies. Further research to support the drug’s effectiveness in managing the symptoms is needed. More studies are needed regarding early diagnosis of NPSLE using the antibodies as biomarkers as it could help in preventing these manifestations.

## Introduction and background

A 42-year-old woman with a 12-year history of seizures and cognitive dysfunction presented with episodes of confusion and psychosis. History was obtained, clinical investigations were done, and it was revealed that she has systemic lupus erythematosus (SLE). Previously, she had mucositis, malar rash, and sunburn; her antinuclear, anti-SM (anti-Smith antibody), and anti-Ro (anti-Sjögren's-syndrome antigen) antibodies were positive. Her erythrocyte sedimentation rate was elevated. The cognitive decline, seizures, and psychosis along with the characteristic symptoms suggest SLE with neuropsychiatric manifestations, and it should be considered in the differential for patients presenting with these features [1].

Systemic lupus erythematosus (SLE) is an autoimmune disease that mainly affects women, typically during the reproductive years. It consists of high titers of autoantibodies in the serum and in numerous end organs leading to various pathologies. It has been found that 25%-75% of patients with SLE report multiple neurological symptoms that are due to the involvement of some parts of the brain and nervous system [2]. Table 1 illustrates a total of 19 syndromes that are associated with NPSLE and are classified by the American College of Rheumatology (ACR) [3].

The neuronal changes are thought to be due to autoantibodies reactive against receptors present in the brain and against several membrane proteins. The involvement of interferons and cytokines has also been suggested. The autoantibodies binding N-methyl-D-aspartate receptor (NMDAR) are being studied in this review. Some of the antibodies involved are given below in Table 2.

**Table 1:** Neuropsychiatric syndromes in SLE as per ACR classification SLE - systemic lupus erythematosus, ACR - American College of Rheumatology.

Peripheral Nervous System	Central Nervous System
Cranial neuropathy	Seizure disorder
Mononeuropathy	Cerebrovascular disease
Polyneuropathy	Aseptic meningitis
Plexopathy	Confusion
Autonomic neuropathy	Cognitive dysfunction
Gullian Barre syndrome	Anxiety
Myasthenia gravis	Headache
	Demyelinating syndrome
	Mood disorder
	Psychosis
	Movement disorder
	Myelopathy

**Table 2:** Autoantibodies in neuropsychiatric SLE SLE - systemic lupus erythematosus

Antibodies
Anti-phospholipid antibody
Anti-ribosomal P antibody
Anti-NMDAR antibody
Anti-nuclear antibody
Anti-Smith antibody
Anti-Ro antibody
Anti-neuronal antibody

Seizures, strokes, and other central nervous system (CNS) symptoms in SLE are associated with antiphospholipid antibodies, anti-ribosomal P antibodies, and anti-N-methyl-D-aspartate (NMDA) receptor antibodies and anti-endothelial cell antibodies. CNS plasticity and synapse conduction are maintained by NMDA receptors, which are ligand-gated ion channels that are required for neuronal function, memory, and cognition. Studies have shown that 40% of SLE patients possess titers of anti-NMDA receptor antibody against NR2A/NR2B subunits of NMDAR in the serum and they are the reason for the cognitive decline. The cerebrospinal fluid (CSF) of half the patients suffering from neuropsychiatric SLE are shown to be containing these antibodies. Entry of these antibodies into the brain is dependent on the blood-brain barrier. NMDA receptor activation is seen in many brain disorders like stroke, seizure, and schizophrenia [4-9]. However, the exact mechanism of the elevation and the relevance of these antibodies in NPSLE is not clearly understood [10]. Therefore, we are conducting this analysis to better understand the correlation between anti-NMDA receptor antibodies and neuropsychiatric changes in SLE and to know whether they can be used to assess the risk of developing NPSLE or as diagnostic markers and also to know their association with the disease severity and prognosis. Also, the usage of rituximab in the disease has been under discussion for a very long time, so an emphasis is laid on its effectiveness.

Method

A systematic search was conducted using the following keywords in the PUBMED database and PubMed Central (PMC) from (05/23/2018 - 06/15/2018) and the search results are as follows: On the keyword titled 1) SLE antibody brain, 505 studies were shown; 2) neuropsychiatric SLE, 1129 results were shown; 3) brain autoantibody, 4893 results were shown; 4) B cells in SLE, 2205 results were shown; 5) NMDAR antibody SLE, 53 results were shown; 6) rituximab in SLE, seven results were shown; 7) antiribosomal P antibody, 24 results were shown; 8) antiphospholipid antibody, 12389 results were shown.

A total of 21,862 results were shown using just the keyword searches. The filters were applied and the studies were limited to clinical trials and review articles, which showed 4621 studies. The studies were again limited to the last 10 years and 1894 results were included. The abstracts and full texts of the results were screened and 578 relevant articles were obtained. Further screening based on human and animal studies was done and 476 articles were retrieved. After further analysis, duplicate and irrelevant articles were excluded and a total of 50 studies were finalized for review. The 50 studies included 12 observational studies and 18 review articles. After excluding unrelated data, 26 studies were finalized for discussion.

Quality assessment (external validity) and ethical issues

All the information collected has been obtained lawfully. Almost all the data included under the study is from PUBMED and PMC and published in scientific journals with a journal impact factor > 1. Most of the data is peer reviewed and all the articles included are publications after the year 2000.

## Review

The different kinds of biomarkers that are involved in causing neurological and psychiatric changes in SLE have been studied and analyzed in this discussion.

NMDA receptor antibodies

Massardo et al. [11] conducted a study in 2014 on 133 Chilean women. Cognitive deficit, spatial planning disabilities, and attention deficit were found in 20% of women and it was found to be the result of anti-NMDAR antibodies and anti-ribosomal p antibodies causing frontoparietal cortex dysfunction [11]. This study is limited to the Chilean women only and much inference cannot be sought.

NMDAR encephalitis has immunoglobulin G (IgG) antibodies against the extracellular N-terminal domain of the NR-1 subunit. It has a characteristic progression that includes an early phase consisting of cognitive dysfunction and seizures, which can later progress to movement disorder, psychosis, and coma. Behavioral and psychiatric symptoms are thought to be present in around 80% of adults who are diagnosed with NMDAR antibody encephalitis. Anxiety, agitations, paranoid thoughts, and memory loss can be found frequently. Hippocampal damage is seen on magnetic resonance imaging (MRI) usually in patients with memory deficits [12]. A meta-analysis conducted by Tay et al. [13] found an association between antibodies against NR2A/NR2B subunits of NMDAR and neuropsychiatric changes in SLE and systemic sclerosis. Seventeen studies were included in the meta-analysis of 2212 SLE patients and 99 systemic sclerosis patients, and 24.6% patients with SLE and 19.4% of systemic sclerosis patients showed the presence of these antibodies in the serum/plasma and a significantly higher correlation was seen with neurological and psychiatric changes [OR=1.607 ] [13]. According to us, this study shows the strongest evidence about the antibody association with the disease as it is a meta-analysis, which is better when compared to any other clinical study, according to the study design pyramid.

According to one study, the percentage of anti NR2A/B antibodies in NPSLE varied in different studies, but in another study it was found that these antibodies were present in 81% of the SLE patients with diffuse NPSLE and in 41% with focal NPSLE [14]. Following a study conducted by Park et al. [15], an interesting correlation between anti NMDAR antibodies against GluN2B subunits and fibromyalgia in SLE patients has been noted [15]. This study gives us an insight into how the NMDAR antibodies are involved in causing various morbidities in SLE patients. Another study stated that anti-dsDNA cross-reacts with anti NMDAR and this antibody is found in 30% of patients with SLE and the correlation was studied in different organs and is found to be significantly higher in NPSLE. It also mentioned that the presence of these antibodies was associated with cytopenias [16].

A review article by Lauvsnes et al. [8] laid emphasis on the dose-dependent action of anti-NR2A antibodies in NPSLE; they cause neuronal cell death at a high dose and at low dose change the synaptic action. They have also stated that among the human studies, six of the 13 showed an association between NPSLE and the antibodies against NR2A subunit receptor [8]. Mader et al. [17] reported that antibodies to different proteins were the basis for autoimmune diseases and these antibodies penetrate the blood-brain barrier (BBB) and cause neurocognitive changes, an example given was of NMDAR antibody in SLE. In utero exposure to some of these antibodies can cross the BBB of the embryo and cause neurodevelopmental impairment [17]. Su et al. [18] stated that anti-complement 1q antibody is associated with kidney injury, anti NMDAR antibody is associated with psychiatric manifestations, and anti-Mullerian hormone antibody is associated with ovarian damage in lupus [18]. This study proves that various antibodies affect different organ systems in SLE.

A review article by González et al. [19] reported a clinical and experimental evidence that revealed the role of anti-ribosomal p antibodies (neuronal surface P antigen) which are shown to cross-react with NMDAR causing diffuse brain manifestations in NPSLE [19]. An excellent review article by Sen Hee et al. [20] states that anti-NR2A/B antibodies are generated extrathecally and cross the BBB to enter into the CSF and affect the brain function. Matrix metalloproteinase leads to the degradation of the basal lamina and BBB integrity is lost. Anti-NR2A/B antibodies stimulate the BBB by increasing the expression of endothelial cell adhesion molecules, which facilitate the recruitment, adhesion, and transmigration of white cells, leading to increased neutrophil extracellular traps (NET) intrathecally. This contributes to neurotoxicity by inducing neuronal cell death, which subsequently causes neuropsychiatric manifestations in SLE [20].

Inflammatory markers

A review article by Wen et al. [21] states the role of B cells in the adverse behavioral symptoms in NPSLE as it crosses the blood-brain barrier and causes glial activation and neurodegeneration [21]. Improper regulation of cytokines IFN-α, tumor necrosis factor (TNF), and IL-6 may cause disruption of the BBB, and also the elevated levels of cytokines within the CNS such as TNF-like weak inducer of apoptosis (TWEAK) may be responsible for some of the neuropsychiatric changes in lupus [22,23]. This study has brought a new dimension in terms of the pathophysiology and diagnostic approach to NPSLE.

A study conducted in 2017 that included 261 subjects and 261 controls measured the levels of IFN-λ1, IFN-α, IL-17A, IL-23, and IP-10 using ELISA (Enzyme-linked immunosorbent assay), and high levels of IFN-λ1 and IFN-α were seen in NPSLE and high levels of IP-10 were seen in arthritis. The IFN-λ1 level also correlates with T-helper type 17 cytokines that cause more damage [24]. Bialas et al. [25] reported behavioral changes and loss of synapses in mice that have lupus, which was reversed when the Type 1 interferon signaling was blocked. Also, it is shown that the IFN stimulates microglia to damage the synaptic neuron. Hippocampal brain section from SLE patients showed increased levels of interferon type 1 [25]. Anifrolumab, a type 1 interferon antagonist is being studied to be used in NPSLE. This is a recent study in pharmacology in NPSLE [25]. The focal symptoms in NPSLE are believed to result from vascular lesions and the diffuse manifestations are thought to be due to the cytokines that are elevated in NPSLE, which again go back to normal levels when the NPSLE is treated. CX3CL1, a chemokine has been shown to be elevated in the cerebrospinal fluid of patients with NPSLE [26]. TWEAK/Fn14 is shown to cause inflammation by activating nuclear factor-κB pathway and mitogen-activated protein kinase signal, which in turn leads to secretion or release of cytokines, matrix metalloproteinase 9, microglia activation, and it is strongly linked to abnormal manifestations of NPSLE [27].

TWEAK and chemokines are the newly found markers and should be studied in other inflammatory and autoimmune disorders. These studies show the association between various inflammatory markers and NPSLE, and they can be used to formulate new diagnostic criteria for faster detection and prophylactic management before lupus affects the brain. 

Antibodies against membrane proteins

A meta-analysis was conducted to measure the levels of antibodies in blood and CSF in NPSLE, and a total of 41 studies were included in the analysis. When compared to SLE patients with no neuropsychiatric issues, a huge number of NPSLE patients showed positive for antiphospholipid antibody (OR=2.08, p=0.001) and for anti-ribosomal P antibodies (OR=2.29, p<0.001) [28]. This study, in our opinion, is better as it has shown greater odds for these antibodies and it should be further evaluated whether these antibodies are useful for diagnostic purposes.

A systematic review analyzed antibodies in NPSLE in a total of 42 studies and found that antiphospholipid antibody (lupus anticoagulant) and anti-ribosomal p antibodies are elevated significantly in NPSLE and specifically cause stroke and psychiatric symptoms, respectively [29]. A meta-analysis conducted by Shi et al. [30] that included 16 cohort studies with 2355 individuals suggests that NPSLE and hepatic involvement in SLE is observed mostly in patients with positive anti-ribosomal P antibodies [30]. According to a case-control study by Gaber et al. [31] anti-ribosomal P antibodies could be used for the diagnosis of CNS neuropathies in SLE [31].

Another study that included 58 patients checked for neurocognitive impairments associated with various antibodies in SLE, and it was concluded that antiphospholipid antibodies are associated with global cognitive dysfunction and, specifically, visuospatial impairments were typically seen with high anticardiolipin IgM antibody levels (p=0.005) [32]. This study is old but very relevant as they have used various domains of cognition and compared it with the different antibodies in SLE. Depression and psychosis in patients with SLE were found to be associated with antibody-mediated thrombosis and vasculopathy, specifically with anti-endothelial antibodies, which are present significantly in the serum and CSF of SLE patients [33]. Some of the pathogenic factors are mentioned in Table 3*.*

**Table 3:** Pathogenic markers theorized in NPSLE TWEAK - tumor necrosis factor-like weak inducer of apoptosis, NPSLE - neuropsychiatric systemic lupus erythematosus.

Markers
NMDAR antibody (NR1/NR2)
Anti-cardolipin, Beta 2 glycoprotein
Anti-ribosomal P
Anti-endothelial receptor
Sm/Ro (SSA)/U1 RNP
Interferons such as IL-2, IL-6, IL-8, IL-10, TNF, IFN-α, IFN-γ
Chemokine like CXCL 10 and CCL 5
Septins
Anti-histone
Inflammatory markers
B cells
Atherosclerosis
Immune complex deposits
TWEAK

Therapeutic drugs

NMDAR antibody encephalitis patients experienced improvement in neuropsychiatric symptoms with immune therapy that includes steroids, plasma exchange, and intravenous (IV) immunoglobulins as the first line and rituximab as the second line [13]. A systematic review conducted in 2017 that included 272 patients suggested that rituximab improved neuropsychiatric, renal, haematologic disease process in pediatric SLE [34]. The results of this study look promising for the treatment of pediatric SLE, and as it is a comparatively new study we can expect advances in therapeutics for NPSLE.

B-cell hyperactivity has been the cause of the production of antibodies in SLE; therefore, therapy to target the B cells are evolving. Anti-CD20 antibody rituximab and anti-CD22 antibody epratuzumab are being used. Clinical trials have stated that rituximab is highly effective in the treatment of lupus nephritis and NPSLE. A newly found monoclonal antibody directed against CD-20 such as ofatumumab is a fully human antibody. Eculizumab inhibits complement activation. Monoclonal antibodies blocking TNF-alpha (tumor necrosis factor) and IL-6 (interleukin 6) have also been shown to play a role in the treatment of NPSLE [35]. Sanna et al. [36] state that IV cyclophosphamide can be used when the symptoms are refractory to steroid treatment. Mycophenolate mofetil, rituximab, and methotrexate are under study, and plasmapheresis can be considered for refractory illness [36].

## Conclusions

This article acknowledges and outlines the mechanisms behind the neuropsychiatric manifestations in patients suffering from SLE. After reviewing all the available and relevant data, the main key points of the review are that there are some specific antibodies such as NMDAR antibody, antiphospholipid antibody, anti-endothelial antibody, anti-ribosomal P antibody, interferons, and TWEAKs that target specific areas of the brain in SLE leading to the classic symptoms seen in the disease process. The article focuses on the pathophysiology and it forms the strength of the paper as noted, it also emphasizes the pharmacology behind the drugs used in the disease and the newer treatment options available. It includes the most recent and relevant studies in the discussion section and all the data have been collected from scientific journals. The paper does have some limitations since there are very few observational and experimental studies conducted on this topic; therefore, we need more studies specifically on the NMDAR antagonism, studies to easily identify and detect the specific biomarkers in the serum/CSF of NPSLE patients, and finally, to identify the best treatment modalities available to treat NPSLE.
